# Supplementary material for: HIVprotI: an integrated web based platform for prediction and design of HIV proteins inhibitors
Source: J Cheminform. 2018 Mar 9;10:12. doi: 10.1186/s13321-018-0266-y (PMC5845081; doi:10.1186/s13321-018-0266-y)
Supplement: Supplementary file 2 — Additional file 2. Source code of HIVProtI web server. [file 13321_2018_266_MOESM2_ESM.zip › HIVprotI_Source-code/hivprotI/weka-3-6-6/doc/weka/core/Capabilities.html]

Capabilities


---


|  |  |  |
| --- | --- | --- |
| **Overview** \| **Package** \| **Class** \| **Tree** \| **Deprecated** \| **Index** \| **Help** \| **Weka's home** | |  |
| **PREV CLASS**   **NEXT CLASS** | **FRAMES**    **NO FRAMES**     **All Classes** |  |
| SUMMARY: NESTED \| FIELD \| CONSTR \| METHOD | DETAIL: FIELD \| CONSTR \| METHOD |  |


---


## weka.core Class Capabilities

```
java.lang.Object
  weka.core.Capabilities
```

**All Implemented Interfaces:**: java.io.Serializable, java.lang.Cloneable, RevisionHandler

---

``` public class Capabilities extends java.lang.Object implements java.lang.Cloneable, java.io.Serializable, RevisionHandler ```

A class that describes the capabilites (e.g., handling certain types of
attributes, missing values, types of classes, etc.) of a specific
classifier. By default, the classifier is capable of nothing. This
ensures that new features have to be enabled explicitly.

A common code fragment for making use of the capabilities in a classifier
would be this:

```
 public void buildClassifier(Instances instances) throws Exception {
   // can the classifier handle the data?
   getCapabilities().testWithFail(instances);
   ...
   // possible deletion of instances with missing class labels, etc.
```

For only testing a single attribute, use this:

```
   ...
   Attribute att = instances.attribute(0);
   getCapabilities().testWithFail(att);
   ...
```

Or for testing the class attribute (uses the capabilities that are
especially for the class):

```
   ...
   Attribute att = instances.classAttribute();
   getCapabilities().testWithFail(att, true);
   ...
```

**Version:**
:   $Revision: 7082 $

**Author:**
:   FracPete (fracpete at waikato dot ac dot nz)

**See Also:**: Serialized Form

---

| **Nested Class Summary** | |
| --- | --- |
| `static class` | `Capabilities.Capability`             enumeration of all capabilities |


| **Field Summary** | |
| --- | --- |
| `static java.lang.String` | `PROPERTIES_FILE`             the properties file for managing the tests |


**Constructor Summary**

`Capabilities(CapabilitiesHandler owner)`
  
          initializes the capabilities for the given owner


| **Method Summary** | |
| --- | --- |
| `void` | `and(Capabilities c)`             performs an AND conjunction with the capabilities of the given Capabilities object and updates itself |
| `void` | `assign(Capabilities c)`             retrieves the data from the given Capabilities object |
| `java.util.Iterator` | `capabilities()`             Returns an Iterator over the stored capabilities |
| `java.lang.Object` | `clone()`             Creates and returns a copy of this object. |
| `java.util.Iterator` | `dependencies()`             Returns an Iterator over the stored dependencies |
| `void` | `disable(Capabilities.Capability c)`             disables the given capability Disabling NOMINAL\_ATTRIBUTES also disables BINARY\_ATTRIBUTES, UNARY\_ATTRIBUTES and EMPTY\_NOMINAL\_ATTRIBUTES. |
| `void` | `disableAll()`             disables all attribute and class types (including dependencies) |
| `void` | `disableAllAttributeDependencies()`             disables all attribute type dependencies |
| `void` | `disableAllAttributes()`             disables all attribute types |
| `void` | `disableAllClassDependencies()`             disables all class type dependencies |
| `void` | `disableAllClasses()`             disables all class types |
| `void` | `disableDependency(Capabilities.Capability c)`             disables the dependency of the given capability Disabling NOMINAL\_ATTRIBUTES also disables BINARY\_ATTRIBUTES, UNARY\_ATTRIBUTES and EMPTY\_NOMINAL\_ATTRIBUTES. |
| `void` | `enable(Capabilities.Capability c)`             enables the given capability. |
| `void` | `enableAll()`             enables all attribute and class types (including dependencies) |
| `void` | `enableAllAttributeDependencies()`             enables all attribute type dependencies |
| `void` | `enableAllAttributes()`             enables all attribute types |
| `void` | `enableAllClassDependencies()`             enables all class type dependencies |
| `void` | `enableAllClasses()`             enables all class types |
| `void` | `enableDependency(Capabilities.Capability c)`             enables the dependency flag for the given capability Enabling NOMINAL\_ATTRIBUTES also enables BINARY\_ATTRIBUTES, UNARY\_ATTRIBUTES and EMPTY\_NOMINAL\_ATTRIBUTES. |
| `static Capabilities` | `forInstances(Instances data)`             returns a Capabilities object specific for this data. |
| `static Capabilities` | `forInstances(Instances data, boolean multi)`             returns a Capabilities object specific for this data. |
| `Capabilities` | `getAttributeCapabilities()`             returns all attribute capabilities |
| `Capabilities` | `getClassCapabilities()`             returns all class capabilities |
| `java.lang.Exception` | `getFailReason()`             returns the reason why the tests failed, is null if tests succeeded |
| `int` | `getMinimumNumberInstances()`             returns the minimum number of instances that have to be in the dataset |
| `Capabilities` | `getOtherCapabilities()`             returns all other capabilities, besides class and attribute related ones |
| `CapabilitiesHandler` | `getOwner()`             returns the owner of this capabilities object |
| `java.lang.String` | `getRevision()`             Returns the revision string. |
| `boolean` | `handles(Capabilities.Capability c)`             returns true if the classifier handler has the specified capability |
| `boolean` | `hasDependencies()`             Checks whether there are any dependencies at all |
| `boolean` | `hasDependency(Capabilities.Capability c)`             returns true if the classifier handler has a dependency for the specified capability |
| `static void` | `main(java.lang.String[] args)`             loads the given dataset and prints the Capabilities necessary to process it. |
| `void` | `or(Capabilities c)`             performs an OR conjunction with the capabilities of the given Capabilities object and updates itself |
| `void` | `setMinimumNumberInstances(int value)`             sets the minimum number of instances that have to be in the dataset |
| `void` | `setOwner(CapabilitiesHandler value)`             sets the owner of this capabilities object |
| `boolean` | `supports(Capabilities c)`             Returns true if the currently set capabilities support at least all of the capabiliites of the given Capabilities object (checks only the enum!) |
| `boolean` | `supportsMaybe(Capabilities c)`             Returns true if the currently set capabilities support (or have a dependency) at least all of the capabilities of the given Capabilities object (checks only the enum!) |
| `boolean` | `test(Attribute att)`             Test the given attribute, whether it can be processed by the handler, given its capabilities. |
| `boolean` | `test(Attribute att, boolean isClass)`             Test the given attribute, whether it can be processed by the handler, given its capabilities. |
| `boolean` | `test(Instances data)`             Tests the given data, whether it can be processed by the handler, given its capabilities. |
| `boolean` | `test(Instances data, int fromIndex, int toIndex)`             Tests a certain range of attributes of the given data, whether it can be processed by the handler, given its capabilities. |
| `void` | `testWithFail(Attribute att)`             tests the given attribute by calling the test(Attribute,boolean) method and throws an exception if the test fails. |
| `void` | `testWithFail(Attribute att, boolean isClass)`             tests the given attribute by calling the test(Attribute,boolean) method and throws an exception if the test fails. |
| `void` | `testWithFail(Instances data)`             tests the given data by calling the test(Instances) method and throws an exception if the test fails. |
| `void` | `testWithFail(Instances data, int fromIndex, int toIndex)`             tests the given data by calling the test(Instances,int,int) method and throws an exception if the test fails. |
| `java.lang.String` | `toSource(java.lang.String objectname)`             turns the capabilities object into source code. |
| `java.lang.String` | `toSource(java.lang.String objectname, int indent)`             turns the capabilities object into source code. |
| `java.lang.String` | `toString()`             returns a string representation of the capabilities |

**Methods inherited from class java.lang.Object**

`equals, getClass, hashCode, notify, notifyAll, wait, wait, wait`

**Field Detail**

### PROPERTIES\_FILE

```
public static final java.lang.String PROPERTIES_FILE
```

:   the properties file for managing the tests

    **See Also:**: Constant Field Values


**Constructor Detail**

### Capabilities

```
public Capabilities(CapabilitiesHandler owner)
```

:   initializes the capabilities for the given owner

    **Parameters:**: `owner` - the object that produced this Capabilities instance


**Method Detail**

### clone

```
public java.lang.Object clone()
```

:   Creates and returns a copy of this object.

    :   **Overrides:**: `clone` in class `java.lang.Object`
    :   **Returns:**: a clone of this object

---


### assign

```
public void assign(Capabilities c)
```

:   retrieves the data from the given Capabilities object

    :   **Parameters:**: `c` - the capabilities object to initialize with

---


### and

```
public void and(Capabilities c)
```

:   performs an AND conjunction with the capabilities of the given
    Capabilities object and updates itself

    :   **Parameters:**: `c` - the capabilities to AND with

---


### or

```
public void or(Capabilities c)
```

:   performs an OR conjunction with the capabilities of the given
    Capabilities object and updates itself

    :   **Parameters:**: `c` - the capabilities to OR with

---


### supports

```
public boolean supports(Capabilities c)
```

:   Returns true if the currently set capabilities support at least all of
    the capabiliites of the given Capabilities object (checks only the enum!)

    :   **Parameters:**: `c` - the capabilities to support at least **Returns:**: true if all the requested capabilities are supported

---


### supportsMaybe

```
public boolean supportsMaybe(Capabilities c)
```

:   Returns true if the currently set capabilities support (or have a
    dependency) at least all of the capabilities of the given Capabilities
    object (checks only the enum!)

    :   **Parameters:**: `c` - the capabilities (or dependencies) to support at least **Returns:**: true if all the requested capabilities are supported (or at least have a dependency)

---


### setOwner

```
public void setOwner(CapabilitiesHandler value)
```

:   sets the owner of this capabilities object

    :   **Parameters:**: `value` - the new owner

---


### getOwner

```
public CapabilitiesHandler getOwner()
```

:   returns the owner of this capabilities object

    :   **Returns:**: the current owner of this capabilites object

---


### setMinimumNumberInstances

```
public void setMinimumNumberInstances(int value)
```

:   sets the minimum number of instances that have to be in the dataset

    :   **Parameters:**: `value` - the minimum number of instances

---


### getMinimumNumberInstances

```
public int getMinimumNumberInstances()
```

:   returns the minimum number of instances that have to be in the dataset

    :   **Returns:**: the minimum number of instances

---


### capabilities

```
public java.util.Iterator capabilities()
```

:   Returns an Iterator over the stored capabilities

    :   **Returns:**: iterator over the current capabilities

---


### dependencies

```
public java.util.Iterator dependencies()
```

:   Returns an Iterator over the stored dependencies

    :   **Returns:**: iterator over the current dependencies

---


### enable

```
public void enable(Capabilities.Capability c)
```

:   enables the given capability.
    Enabling NOMINAL\_ATTRIBUTES also enables BINARY\_ATTRIBUTES,
    UNARY\_ATTRIBUTES and EMPTY\_NOMINAL\_ATTRIBUTES.
    Enabling BINARY\_ATTRIBUTES also enables UNARY\_ATTRIBUTES and
    EMPTY\_NOMINAL\_ATTRIBUTES.
    Enabling UNARY\_ATTRIBUTES also enables EMPTY\_NOMINAL\_ATTRIBUTES.
    But NOMINAL\_CLASS only enables BINARY\_CLASS, since normal schemes in Weka
    don't work with datasets that have only 1 class label (or none).

    :   **Parameters:**: `c` - the capability to enable

---


### enableDependency

```
public void enableDependency(Capabilities.Capability c)
```

:   enables the dependency flag for the given capability
    Enabling NOMINAL\_ATTRIBUTES also enables BINARY\_ATTRIBUTES,
    UNARY\_ATTRIBUTES and EMPTY\_NOMINAL\_ATTRIBUTES.
    Enabling BINARY\_ATTRIBUTES also enables UNARY\_ATTRIBUTES and
    EMPTY\_NOMINAL\_ATTRIBUTES.
    Enabling UNARY\_ATTRIBUTES also enables EMPTY\_NOMINAL\_ATTRIBUTES.
    But NOMINAL\_CLASS only enables BINARY\_CLASS, since normal schemes in Weka
    don't work with datasets that have only 1 class label (or none).

    :   **Parameters:**: `c` - the capability to enable the dependency flag for

---


### enableAllClasses

```
public void enableAllClasses()
```

:   enables all class types

    :   **See Also:**: `disableAllClasses()`, `getClassCapabilities()`

---


### enableAllClassDependencies

```
public void enableAllClassDependencies()
```

:   enables all class type dependencies

    :   **See Also:**: `disableAllClassDependencies()`, `getClassCapabilities()`

---


### enableAllAttributes

```
public void enableAllAttributes()
```

:   enables all attribute types

    :   **See Also:**: `disableAllAttributes()`, `getAttributeCapabilities()`

---


### enableAllAttributeDependencies

```
public void enableAllAttributeDependencies()
```

:   enables all attribute type dependencies

    :   **See Also:**: `disableAllAttributeDependencies()`, `getAttributeCapabilities()`

---


### enableAll

```
public void enableAll()
```

:   enables all attribute and class types (including dependencies)

---


### disable

```
public void disable(Capabilities.Capability c)
```

:   disables the given capability
    Disabling NOMINAL\_ATTRIBUTES also disables BINARY\_ATTRIBUTES,
    UNARY\_ATTRIBUTES and EMPTY\_NOMINAL\_ATTRIBUTES.
    Disabling BINARY\_ATTRIBUTES also disables UNARY\_ATTRIBUTES and
    EMPTY\_NOMINAL\_ATTRIBUTES.
    Disabling UNARY\_ATTRIBUTES also disables EMPTY\_NOMINAL\_ATTRIBUTES.
    The same hierarchy applies to the class capabilities.

    :   **Parameters:**: `c` - the capability to disable

---


### disableDependency

```
public void disableDependency(Capabilities.Capability c)
```

:   disables the dependency of the given capability
    Disabling NOMINAL\_ATTRIBUTES also disables BINARY\_ATTRIBUTES,
    UNARY\_ATTRIBUTES and EMPTY\_NOMINAL\_ATTRIBUTES.
    Disabling BINARY\_ATTRIBUTES also disables UNARY\_ATTRIBUTES and
    EMPTY\_NOMINAL\_ATTRIBUTES.
    Disabling UNARY\_ATTRIBUTES also disables EMPTY\_NOMINAL\_ATTRIBUTES.
    The same hierarchy applies to the class capabilities.

    :   **Parameters:**: `c` - the capability to disable the dependency flag for

---


### disableAllClasses

```
public void disableAllClasses()
```

:   disables all class types

    :   **See Also:**: `enableAllClasses()`, `getClassCapabilities()`

---


### disableAllClassDependencies

```
public void disableAllClassDependencies()
```

:   disables all class type dependencies

    :   **See Also:**: `enableAllClassDependencies()`, `getClassCapabilities()`

---


### disableAllAttributes

```
public void disableAllAttributes()
```

:   disables all attribute types

    :   **See Also:**: `enableAllAttributes()`, `getAttributeCapabilities()`

---


### disableAllAttributeDependencies

```
public void disableAllAttributeDependencies()
```

:   disables all attribute type dependencies

    :   **See Also:**: `enableAllAttributeDependencies()`, `getAttributeCapabilities()`

---


### disableAll

```
public void disableAll()
```

:   disables all attribute and class types (including dependencies)

---


### getClassCapabilities

```
public Capabilities getClassCapabilities()
```

:   returns all class capabilities

    :   **Returns:**: all capabilities regarding the class **See Also:**: `enableAllClasses()`, `disableAllClasses()`

---


### getAttributeCapabilities

```
public Capabilities getAttributeCapabilities()
```

:   returns all attribute capabilities

    :   **Returns:**: all capabilities regarding attributes **See Also:**: `enableAllAttributes()`, `disableAllAttributes()`

---


### getOtherCapabilities

```
public Capabilities getOtherCapabilities()
```

:   returns all other capabilities, besides class and attribute related ones

    :   **Returns:**: all other capabilities, besides class and attribute related ones

---


### handles

```
public boolean handles(Capabilities.Capability c)
```

:   returns true if the classifier handler has the specified capability

    :   **Parameters:**: `c` - the capability to test **Returns:**: true if the classifier handler has the capability

---


### hasDependency

```
public boolean hasDependency(Capabilities.Capability c)
```

:   returns true if the classifier handler has a dependency for the specified
    capability

    :   **Parameters:**: `c` - the capability to test **Returns:**: true if the classifier handler has a dependency for the capability

---


### hasDependencies

```
public boolean hasDependencies()
```

:   Checks whether there are any dependencies at all

    :   **Returns:**: true if there is at least one dependency for a capability

---


### getFailReason

```
public java.lang.Exception getFailReason()
```

:   returns the reason why the tests failed, is null if tests succeeded

    :   **Returns:**: the reason why the tests failed

---


### test

```
public boolean test(Attribute att)
```

:   Test the given attribute, whether it can be processed by the handler,
    given its capabilities. The method assumes that the specified attribute
    is not the class attribute.

    :   **Parameters:**: `att` - the attribute to test **Returns:**: true if all the tests succeeded **See Also:**: `test(Attribute, boolean)`

---


### test

```
public boolean test(Attribute att,
                    boolean isClass)
```

:   Test the given attribute, whether it can be processed by the handler,
    given its capabilities.

    :   **Parameters:**: `att` - the attribute to test: `isClass` - whether this attribute is the class attribute **Returns:**: true if all the tests succeeded **See Also:**: `m_AttributeTest`

---


### test

```
public boolean test(Instances data)
```

:   Tests the given data, whether it can be processed by the handler,
    given its capabilities. Classifiers implementing the
    `MultiInstanceCapabilitiesHandler` interface are checked
    automatically for their multi-instance Capabilities (if no bags, then
    only the bag-structure, otherwise only the first bag).

    :   **Parameters:**: `data` - the data to test **Returns:**: true if all the tests succeeded **See Also:**: `test(Instances, int, int)`

---


### test

```
public boolean test(Instances data,
                    int fromIndex,
                    int toIndex)
```

:   Tests a certain range of attributes of the given data, whether it can be
    processed by the handler, given its capabilities. Classifiers
    implementing the `MultiInstanceCapabilitiesHandler` interface
    are checked automatically for their multi-instance Capabilities (if no
    bags, then only the bag-structure, otherwise only the first bag).

    :   **Parameters:**: `data` - the data to test: `fromIndex` - the range of attributes - start (incl.): `toIndex` - the range of attributes - end (incl.) **Returns:**: true if all the tests succeeded **See Also:**: `MultiInstanceCapabilitiesHandler`, `m_InstancesTest`, `m_MissingValuesTest`, `m_MissingClassValuesTest`, `m_MinimumNumberInstancesTest`

---


### testWithFail

```
public void testWithFail(Attribute att)
                  throws java.lang.Exception
```

:   tests the given attribute by calling the test(Attribute,boolean) method
    and throws an exception if the test fails. The method assumes that the
    specified attribute is not the class attribute.

    :   **Parameters:**: `att` - the attribute to test **Throws:**: `java.lang.Exception` - in case the attribute doesn't pass the tests **See Also:**: `test(Attribute,boolean)`

---


### testWithFail

```
public void testWithFail(Attribute att,
                         boolean isClass)
                  throws java.lang.Exception
```

:   tests the given attribute by calling the test(Attribute,boolean) method
    and throws an exception if the test fails.

    :   **Parameters:**: `att` - the attribute to test: `isClass` - whether this attribute is the class attribute **Throws:**: `java.lang.Exception` - in case the attribute doesn't pass the tests **See Also:**: `test(Attribute,boolean)`

---


### testWithFail

```
public void testWithFail(Instances data,
                         int fromIndex,
                         int toIndex)
                  throws java.lang.Exception
```

:   tests the given data by calling the test(Instances,int,int) method and
    throws an exception if the test fails.

    :   **Parameters:**: `data` - the data to test: `fromIndex` - the range of attributes - start (incl.): `toIndex` - the range of attributes - end (incl.) **Throws:**: `java.lang.Exception` - in case the data doesn't pass the tests **See Also:**: `test(Instances,int,int)`

---


### testWithFail

```
public void testWithFail(Instances data)
                  throws java.lang.Exception
```

:   tests the given data by calling the test(Instances) method and throws
    an exception if the test fails.

    :   **Parameters:**: `data` - the data to test **Throws:**: `java.lang.Exception` - in case the data doesn't pass the tests **See Also:**: `test(Instances)`

---


### toString

```
public java.lang.String toString()
```

:   returns a string representation of the capabilities

    :   **Overrides:**: `toString` in class `java.lang.Object`
    :   **Returns:**: a string representation of this object

---


### toSource

```
public java.lang.String toSource(java.lang.String objectname)
```

:   turns the capabilities object into source code. The returned source code
    is a block that creates a Capabilities object named 'objectname' and
    enables all the capabilities of this Capabilities object.

    :   **Parameters:**: `objectname` - the name of the Capabilities object being instantiated **Returns:**: the generated source code

---


### toSource

```
public java.lang.String toSource(java.lang.String objectname,
                                 int indent)
```

:   turns the capabilities object into source code. The returned source code
    is a block that creates a Capabilities object named 'objectname' and
    enables all the capabilities of this Capabilities object.

    :   **Parameters:**: `objectname` - the name of the Capabilities object being instantiated: `indent` - the number of blanks to indent **Returns:**: the generated source code

---


### forInstances

```
public static Capabilities forInstances(Instances data)
                                 throws java.lang.Exception
```

:   returns a Capabilities object specific for this data. The multi-instance
    capability is not checked as well as the minimum number of instances
    is not set.

    :   **Parameters:**: `data` - the data to base the capabilities on **Returns:**: a data-specific capabilities object **Throws:**: `java.lang.Exception` - in case an error occurrs, e.g., an unknown attribute type

---


### forInstances

```
public static Capabilities forInstances(Instances data,
                                        boolean multi)
                                 throws java.lang.Exception
```

:   returns a Capabilities object specific for this data. The minimum number
    of instances is not set, the check for multi-instance data is optional.

    :   **Parameters:**: `data` - the data to base the capabilities on: `multi` - if true then the structure is checked, too **Returns:**: a data-specific capabilities object **Throws:**: `java.lang.Exception` - in case an error occurrs, e.g., an unknown attribute type

---


### main

```
public static void main(java.lang.String[] args)
                 throws java.lang.Exception
```

:   loads the given dataset and prints the Capabilities necessary to
    process it.

    Valid parameters:

    -file filename

---


### getRevision

```
public java.lang.String getRevision()
```

:   Returns the revision string.

    :   **Specified by:**: `getRevision` in interface `RevisionHandler`
    :   **Returns:**: the revision


---


|  |  |  |
| --- | --- | --- |
| **Overview** \| **Package** \| **Class** \| **Tree** \| **Deprecated** \| **Index** \| **Help** \| **Weka's home** | |  |
| **PREV CLASS**   **NEXT CLASS** | **FRAMES**    **NO FRAMES**     **All Classes** |  |
| SUMMARY: NESTED \| FIELD \| CONSTR \| METHOD | DETAIL: FIELD \| CONSTR \| METHOD |  |


---
